# Validation of the specialized competency framework for pharmacists in hospital settings (SCF–PHS): a cross-sectional study

**DOI:** 10.1186/s40545-023-00592-7

**Published:** 2023-07-10

**Authors:** Nibal Chamoun, Elsy Ramia, Hala Sacre, Mansour Haddad, Chadia Haddad, Aline Hajj, Joya Namnoum, Rony M. Zeenny, Katia Iskandar, Marwan Akel, Pascale Salameh

**Affiliations:** 1 Department of Pharmacy Practice, Lebanese American University School of Pharmacy, Byblos, Lebanon; 2 INSPECT-LB (Institut National de Santé Publique, d’Épidémiologie Clinique et de Toxicologie-Liban), Beirut, Lebanon; 3 Drug Information Center, Order of Pharmacists of Lebanon, Beirut, Lebanon; 4 Faculty of Pharmacy, Yarmouk University, Irbid, 21163 Jordan; 5 School of Medicine, Lebanese American University, Byblos, Lebanon; 6 Research Department, Psychiatric Hospital of the Cross, P.O. Box 60096, Jal Eddib, Lebanon; 7 School of Health Sciences, Modern University for Business and Science, Beirut, Lebanon; 8 Laboratoire de Pharmacologie, Pharmacie Clinique et Contrôle de Qualité des Médicament (LPCQM), Faculty of Pharmacy, Saint Joseph University of Beirut, Beirut, Lebanon; 9 Faculté de Pharmacie, Université Laval, Quebec, Canada; 10 Oncology Division, CHU de Québec Université Laval Research Center, Quebec, Canada; 11 Methodology and Statistics in Biomedical Research Unit, Faculty of Medicine, Paris-Saclay University, Kremlin-Bicêtre, Paris, France; 12 Department of Pharmacy, American University of Beirut Medical Center, Beirut, Lebanon; 13 School of Pharmacy, Lebanese International University, Beirut, Lebanon; 14 Faculty of Pharmacy, Lebanese University, Hadat, Lebanon; 15 School of Education, Lebanese International University, Beirut, Lebanon; 16 Department of Primary Care and Population Health, University of Nicosia Medical School, 2417 Nicosia, Cyprus

**Keywords:** Clinical pharmacist, Competency framework, Hospital pharmacist, Specialized competency, Tool validation

## Abstract

**Objectives:**

This study aimed to validate the content of the specialized competency frameworks for pharmacists working in hospital settings (hospital and clinical pharmacists) and pilot the frameworks for practice assessment.

**Methods:**

This online cross-sectional study was carried out between March and October 2022 among a sample of 96 Lebanese pharmacists working in hospital settings. The frameworks were distributed to full-time hospital and clinical pharmacists, who filled them out according to their role in the hospital.

**Results:**

Overall, the competencies were distributed over five domains for hospital pharmacists (fundamental skills, safe and rational use of medicines, patient-centered care, professional skills, and preparedness for emergencies), while for clinical pharmacists, competencies were distributed over seven domains (quality improvement, clinical knowledge and skills, soft skills, ability to conduct clinical research, ability to provide effective education, use information technology to make decisions and reduce errors, and emergency preparedness). Moreover, Cronbach alpha values were appropriate, indicating sufficient to high internal consistency. Pharmacists were highly confident in most competencies, with some exceptions related to research in emergency settings (data evaluation, research, and reporting).

**Conclusions:**

This study could validate competency frameworks for clinical and hospital pharmacists, with the competencies and their respective behaviors showing an adequate construct analysis. It also identified the domains that require further development, i.e., soft skills and research in emergency settings. Both these domains are timely and needed to overcome the current practice challenges in Lebanon.

**Supplementary Information:**

The online version contains supplementary material available at 10.1186/s40545-023-00592-7.

## Introduction

For many decades, hospital and clinical pharmacy services have been growing, benefiting from pharmacists with extra training and expertise to improve aspects of pharmaceutical care in hospitals [1]. Decentralization of services was promoted, and the participation of clinical pharmacists in a structured clinical multidisciplinary team was related to a decrease in hospital admissions [2, 3]. Several countries have adopted such services, while pharmacy professional organizations have emphasized the need to define the competencies and further training necessary for advanced practice and recognize specializations within the field [4, 5]. Components of advanced training and experience are required to practice successfully as clinical or hospital pharmacists.

Clinical pharmacists offer patients in all healthcare settings complete drug management and associated care. They are highly qualified professionals who have received extensive education and training. They possess the clinical skills necessary to work in healthcare settings, where teamwork and direct patient care are emphasized. Their responsibilities go beyond just dispensing medication, as they also monitor for potential drug interactions, provide patient counseling, and work with other healthcare professionals to create personalized treatment plans [6]. As patients move through various healthcare settings and interact with different healthcare providers, it is vital to ensure that their treatment is coordinated and effective. Clinical pharmacists are trained to assess patients, evaluate their medication therapy, and develop and implement individualized plans of care. They also play a critical role in monitoring patients’ medication use over time to ensure that treatment remains safe and effective [7].

As for hospital pharmacists, they have a critical role in patient care that goes beyond the traditional tasks of medication preparation and dispensing. They also contribute to disease management, medication review, and health promotion [5, 8]. Research has shown that the involvement of hospital pharmacists in prescribing and medication management can lead to improved outcomes, including reduced medication errors and adverse drug events [9–11]. In many countries, there is a growing recognition of the benefits of increased collaboration among healthcare professionals in making prescription choices, and the expanded role of pharmacists in prescribing has been particularly effective [12, 13]. Hospital pharmacists provide various types of patient care support, such as recommendations on medication use and storage, medication substitution, and participation in clinical studies involving individualized, high-tech medications [14].

Furthermore, pharmacists who wish to participate in pharmacy practice research and clinical research must possess the knowledge and skills to describe, explain, and discuss commonly used research methodology. They must also be able to organize, direct, and conduct research and practice development programs that promote the safe and responsible use of medications while also collaborating with other healthcare professionals [4, 15]. Several minimum and specialized competency standards/frameworks have been established beyond those required by formal training programs to ensure that pharmacists possess the necessary competencies to work effectively in these roles. These standards encompass a range of behavioral competencies and knowledge elements, each with its own set of outcomes. By meeting these standards, pharmacists can work safely and competently in research and practice development, helping to advance the pharmacy profession and improve patient care [16–19].

In Lebanon, few pharmacists choose clinical/hospital pharmacy as a career path due to the paucity of positions and the fact that these specialties are not recognized by local authorities [20, 21]. Although schools of pharmacy provide various didactic and clinical hours in patient care, the exposure to such programs varies, affecting the knowledge, abilities, and perspectives of new graduates when it comes to providing patient care. Since there is a smaller number of pharmacists with formal training in hospitals, hospital pharmacists often need significant on-the-job training to attain a basic level of proficiency in caring for patients, in addition to training aimed at developing broad clinical and organizational abilities relevant to all patient demographics and pharmacy environments [22]. Before engaging in an unsupervised practice, pharmacists providing patient care in a hospital setting must demonstrate competence in fundamental knowledge and abilities. Pharmacists who have not completed hospital residency training may need to attain this basic level of proficiency through institution-based on-the-job training programs [23, 24].

Currently, there are no established minimum competency requirements for entry-level pharmacists who work in hospital settings. The Order of Pharmacists of Lebanon (OPL), the professional association of pharmacists in Lebanon, had issued a core competencies framework to improve the quality, safety, and equality of patient care in Lebanon [24, 25]. It has also developed guiding principles for service reform of clinical/hospital pharmacists, which includes suggested frameworks for academic institutions to incorporate into their curricula and for healthcare institutions to use in service redesign [23]. The subsequent pharmaceutical reform should be patient-centered, engage all relevant parties, encourage effective teamwork, and enhance the patient experience and response. Of note, these suggested that frameworks have not been validated or assessed in practice settings.

Therefore, this study aimed to validate the content of the specialized competency frameworks for pharmacists (hospital and clinical) working in hospital settings (SCF–PHS) and pilot the frameworks for practice assessment.

## Methods

### Source of the competency frameworks

The two frameworks were built upon previous frameworks suggested by the OPL [23]. In addition, given the challenging socioeconomic and sanitary crisis in Lebanon during the current assessment, the authors included a section on emergency preparedness, customized to the responsibilities of pharmacists in hospital settings [26–29].

### Content validation of the frameworks

A panel of six pharmacy experts with academic backgrounds assessed the content of the frameworks. The panel consisted of two hospital pharmacists and four clinical pharmacists; all experts were also researchers. A Delphi technique was applied to agree by more than 90% on the suggested items until reaching a consensus [30, 31]. The final frameworks were finalized and created on Google Forms for diffusion and subsequent assessment.

### Piloting the competency frameworks

This online cross-sectional study was carried out between March and October 2022. The frameworks were distributed to full-time hospital and clinical pharmacists in Lebanon (overall estimated *n* = 150). Pharmacists completed the questionnaire according to their role in the hospital (hospital or clinical). Respondents were briefed about the topic and the different aspects of the questionnaire before filling it out in the introductory section of the questionnaire. Each framework required approximately 30 min to be completed.

### Ethics approval

The Lebanese International University Ethics and Research Committee approved the project (Approval number: 2020RC-063-LIUSOP). Before enrolling in the survey, participants were required to read and consent to the study objectives and the average expected time to complete the questionnaire. Participation was voluntary, and pharmacists received no incentive in return for their participation. No follow-up was possible as data were collected anonymously.

### Sample size calculation

The CDC Epi-info software was used to calculate the minimum sample size using the [32]. The frequency was set at 90%, since the specialized competencies and domains were expected to be fulfilled by working pharmacists. Accordingly, based on a total of 150 pharmacists in hospital settings, a minimum sample size of 93 participants was required to produce an acceptable error of 5%, an expected level of competency confidence of 80%, and a 95% confidence interval, with a 5% alpha error and a power of 80%. Data collection was stopped when 96 pharmacists completed the frameworks.

### Description of the questionnaires

The questionnaires were available in English, because it is a widely spoken language among health professionals in Lebanon, including pharmacists in hospital settings (Additional files 1 and 2).

The first section covered sociodemographic, educational, and professional characteristics. In this part of the questionnaire, participants were asked about several features, e.g., age, gender, nationality, area of work, university of graduation, highest educational level, years of experience, and the number of days of work per week.

The second part of the questionnaire consisted of the respective frameworks. Domains included competencies and behaviors (one question per item) related to hospital or clinical pharmacy practice. Behaviors were rated on a 5-point Likert scale from highly confident (5 points) to fairly confident (4 points), not sure/I do not know (3 points), slightly confident (2 points), and not confident at all (1 point). Competency scores were calculated by summing the answers to items (behaviors); answers were standardized over one hundred for ease of comparison.

### Statistical analysis

The data were analyzed using SPSS software version 25. A descriptive analysis was performed using counts and percentages for categorical variables and means (M) and standard deviations (SD) for continuous measures, in addition to median and interquartile range (IQR). Bivariate analysis was conducted using nonparametric tests, since the normality of continuous variables was not ensured; the Mann–Whitney test was used to compare two groups, and the ANOVA was used to compare three groups or more. A *p* value of more than 0.05 would be considered significant.

## Results

A total of 96 pharmacists (86 hospital pharmacists and 10 clinical pharmacists), who graduated from all universities in Lebanon, participated in this study. Regarding hospital pharmacists, 83.9% were females, and 35% had a BS Pharmacy. In contrast, 70% of clinical pharmacists were females, and all had a Doctor of Pharmacy degree from the Lebanese American University (LAU) or the Saint Joseph University of Beirut (USJ). Only clinical pharmacists provided direct patient care. Around 40% of participants had another field of work, such as academia and community pharmacy practice. Finally, hospital pharmacists had more years of experience than clinical pharmacists (Table 1).

**Table 1:** Sociodemographic and other characteristics of the pharmacists

	Hospital pharmacists (*N* = 86)	Clinical pharmacists (*N* = 10)
	Frequency (%)	Frequency (%)
Gender		
Male	14 (16.1%)	3 (30.0%)
Female	73 (83.9%)	7 (70.0%)
Level of education*		
BS Pharmacy	79 (90.8%)	7 (70.0%)
PharmD/DPharm	47 (54.0%)	10 (100%)
Masters	23 (26.4%)	7 (70.0%)
PhD	2 (2.3%)	0 (0%)
Highest degree related to your main field of work		
BS Pharmacy	31 (35.6%)	–
PharmD/DPharm	37 (42.5%)	4 (40.0%)
Master’s degree	15 (17.2%)	5 (50.0%)
University diploma in clinical and hospital pharmacy	1 (1.1%)	–
PhD	2 (2.3%)	
Postgraduate diplomas	1 (1.1%)	
Other	–	1 (10.0%)
University of graduation as a pharmacist		
LIU	24 (27.6%)	–
UL	24 (27.6%)	–
USJ	13 (14.9%)	6 (60.0%)
BAU	11 (12.6%)	–
LAU	11 (12.6%)	4 (40.0%)
Other	4 (4.6%)	–
University of the highest earned degree		
UL	24 (27.9%)	1 (10.0%)
LIU	22 (25.6%)	–
LAU	11 (12.8%)	2 (20.0%)
BAU	10 (11.6%)	-
USJ	9 (10.5%)	5 (50.0%)
Other	10 (11.6%)	2 (20.0%)
Language of pharmacy education		
French	30 (34.5%)	6 (60.0%)
English	56 (64.4%)	4 (40.0%)
Both	1 (1.1%)	
Work location		
Beirut	34 (39.1%)	8 (80.0%)
Mount Lebanon	21 (24.1%)	2 (20.0%)
North	11 (12.6%)	–
South	12 (13.8%)	
Beqaa	9 (10.3%)	
Number of beds at the hospital		
< 50	18 (20.7%)	
50–100	31 (35.6%)	2 (20.0%)
101–300	29 (33.3%)	5 (50.0%)
> 300	9 (10.3%)	3 (30.0%)
Providing direct patient care		
On Rounds	–	6 (60.0%)
Telehealth (Zoom, Webex, phone calls)	–	0
Both	–	4 (40.0%)
Having another field of work		
I do not have another field of work	51 (58.6%)	6 (60.0%)
Clinical pharmacy	12 (13.8%)	–
Academia (teaching; precepting)	15 (17.2%)	4 (40.0%)
Community pharmacists	6 (6.9%)	–
Other	3 (3.4%)	–

	Mean ± SD	Mean ± SD
Age	35.49 ± 10.49	27.90 ± 4.45
Number of working days per week	5.03 ± 1.19	5.30 ± 0.48
Number of working hours per day	7.85 ± 2.26	8.60 ± 0.69
Years of experience	9.97 ± 10.92	4.42 ± 4.43

*Each participant might have several responses

*BAU* Beirut Arab University, *LAU* Lebanese American University, *LIU* Lebanese International University, *UL* Lebanese University, *USJ* Saint Joseph University of Beirut

Table 2 presents the factor analysis of the competencies and their related behaviors, showing adequate construct analysis. Cronbach alpha values were appropriate, indicating sufficient to high internal consistency. Overall, the competencies were distributed over five domains, i.e., fundamental skills, safe and rational use of medicines, patient-centered care, professional skills, and preparedness for emergencies (Table 2).

**Table 2:** Factor analysis of Lebanese hospital pharmacists’ competencies (Promax rotated component matrix)

Competency		Loading	Cronbach alpha
Domain 0: Fundamental skills			
0.1 Regulations			
1	Ensure prescriptions meet national legal requirements for medicines and medicinal products	0.835	0.781
2	Ensure medicines are labelled appropriately and comply with legislation and national guidance	0.798	
3	Follow hospital regulations pertaining to the operations of the hospital pharmacy	0.783	
4	Apply pharmaceutical statutory regulations	0.702	
5	Demonstrate knowledge of the employment laws	0.608	
Note: Kaiser–Meyer–Olkin (KMO) 0.713; Bartlett’s test of sphericity < 0.001; Percentage of variance explained 56.19%			
0.2 Drug Procurement and Management			
1	Acquire negotiation skills for drug procurement	0.847	0.896
2	Learn the complexity of the drug and medical supplies market	0.804	
3	Supervise procurement systems	0.790	
4	Handle drug shortages and suggest suitable alternatives of medicines and medical devices in a timely manner	0.787	
5	Handle drug waste	0.751	
6	Manage backorders and recalls	0.737	
7	Consider cost-effectiveness when purchasing and dispensing stock and advising on prescribing choices	0.713	
8	Ensure drug inventory management, including tracking of expiry dates, production lot numbers, and storage conditions	0.655	
9	Select medications and develop a therapeutic formulary according the international and national guidelines	0.582	
Kaiser–Meyer–Olkin (KMO) 0.888; Bartlett’s test of sphericity < 0.001; Percentage of variance explained 55.46%			
0.3. Optimize Processes Related to Organization, Medication Preparation, and Delivery			
1	Describe the appropriate roles of pharmacy staff and pharmacists in these processes	0.866	0.676
2	Supervise pharmacy staff in their work in medication preparation and delivery	0.815	
3	Demonstrate organizational skills and develop standard operating procedures related to storage, safety, prescription, preparation, transcription, dispensing, administration, and monitoring steps	0.670	
Kaiser–Meyer–Olkin (KMO) 0.605; Bartlett’s test of sphericity < 0.001; Percentage of variance explained 62.18%			
0.4. Aseptic Techniques			
1	Follow aseptic and sterilization techniques and describe processes and facilities needed to provide sterile compounded parenteral solutions, including the basic requirements of hospital accreditation standards	0.901	0.880
2	Develop standards and maintain written procedures for sterile and aseptic production of medicines, and total parental nutrition	0.899	
3	Apply knowledge of hospital hygiene and infection prevention control (IPC)	0.897	
Kaiser–Meyer–Olkin (KMO) 0.746; Bartlett’s test of sphericity < 0.001; Percentage of variance explained 80.88%			
0.5. Pharmaceutical and Hospital Technology/Automation			
1	Understand their appropriate and safe use as well as unintended consequences	0.928	0.889
2	Outline the basic functionality of commonly used automated systems related to medication use (such as automated dispensing cabinets, computerized prescriber order entry systems, bar code, medication administration systems, programmable infusion devices, and robotics)	0.909	
3	Ensure the effective introduction of new technologies	0.890	
Kaiser–Meyer–Olkin (KMO) 0.739; Bartlett’s test of sphericity < 0.001; Percentage of variance explained 82.66%			
0.6. Pharmaceutical/Medical Skills			
1	Integrate and interface the clinical and distributive functions, including the synergy that translates into safe and effective medication therapy	0.867	0.891
2	Integrate pharmaceutical oncology, nutrition, and other fields when applicable	0.849	
3	Appraise the inter-relationships between formulation (including excipients), drug delivery and therapeutic product	0.835	
4	Participate in designing and implementing pharmaceutical/therapeutic protocols and algorithms	0.780	
5	Demonstrate knowledge of pharmaceutical radiotherapy: therapeutic and diagnostic applications (e.g., contrasts) when applicable	0.753	
6	Describe the use of medical devices, prostheses, and implants when applicable	0.704	
7	Implement the appropriate use of injectable medications, including intravenous, intrathecal, intraocular, intradermal, and other routes	0.686	
8	Formulate, reconstitute and compound medicines when needed	0.677	
9	Develop protocols for ensuring quality of prepared medicines	0.601	
Kaiser–Meyer–Olkin (KMO) 0.857; Bartlett’s test of sphericity < 0.001; Percentage of variance explained 57.01%			
0.7. Business management skills			
1	Perform financial management	0.960	0.947
2	Set budgeting proposals	0.928	
3	Perform accounting activities	0.917	
4	Manage the payment terms and schedules with the suppliers	0.913	
Kaiser–Meyer–Olkin (KMO) 0.792; Bartlett’s test of sphericity < 0.001; Percentage of variance explained 86.04%			
Domain 1: Safe and Rational Use of Medicines			
1.1. Patient Safety			
1	Manage the problems related to switch patients medication to formulary medicines	0.848	0.896
2	Employ performance improvement techniques used in health systems and describe how they are used to appropriate communications to pharmacy providers involved	0.816	
3	Describe the impact of pharmacist involvement on medication safety and quality using appropriate literature	0.812	
4	Reconcile effectively the medications of a patient transitioning from one care setting to another and make appropriate communications to involved pharmacy providers	0.778	
5	Understand patient safety culture that relates to medication use, pharmaceutical care, and pharmacists role	0.771	
6	Implement and promote pharmacovigilance activities within the hospital	0.767	
7	Report and document patient safety incidents	0.752	
Kaiser–Meyer–Olkin (KMO) 0.874; Bartlett’s test of sphericity < 0.001; Percentage of variance explained 62.82%			
1.2. Quality assurance			
1	Discuss with and advise healthcare providers on issues related to pharmaceutical products and quality standards	0.880	0.774
2	Describe the quality standard of the accreditation bodies	0.833	
3	Apply national standards, guidelines, best practices, and established principles and processes related to quality and safe medication use (e.g., storage of look-alike/sound-alike medications, high alert medications, storage of concentrated potassium in patient care areas, dangerous abbreviations, leading decimal points and trailing zeros, quality measures related to medications, etc.)	0.713	
4	Identify storage conditions and secure the medicines cold chain conditions	0.697	
Kaiser–Meyer–Olkin (KMO) 0.697; Bartlett’s test of sphericity < 0.001; Percentage of variance explained 61.59%			
1.3. Pharmacovigilance			
1	Identify a potential adverse drug reaction	0.931	0.761
2	Plan and implement medicines management actions to minimize the risk related to these medicines	0.815	
3	Consider that reporting an ADR is part of pharmacist duties	0.719	
Kaiser–Meyer–Olkin (KMO) 0.529; Bartlett’s test of sphericity < 0.001; Percentage of variance explained 68.27%			
Domain 2: Patient-centered care			
2.1. Literature Evaluation/Search/Trials			
1	Read recently published studies critically	0.870	0.846
2	Develop and implement methods for clinical trials and observational studies	0.858	
3	Provide accurate and evidence-based answers	0.858	
4	Access appropriate drug information resources, including primary literature	0.779	
Kaiser–Meyer–Olkin (KMO) 0.764; Bartlett’s test of sphericity < 0.001; Percentage of variance explained 70.92%			
2.2. Pharmacokinetic-Based Assessment			
1	Evaluate drug-response and monitor patients	0.950	0.949
2	Evaluate medication-use patterns in a specified patient population	0.948	
3	List the medications that need pharmacokinetic evaluation	0.918	
4	Apply dosing principle	0.909	
Kaiser–Meyer–Olkin (KMO) 0.805; Bartlett’s test of sphericity < 0.001; Percentage of variance explained 86.79%			
2.3. Drug Use Optimization			
1	Optimize use of drugs, including addition, deletion, dose adjustment, intravenous to oral route switch, renal dosing, dose reduction, etc.	0.852	0.861
2	Draft and distribute information and recommendations	0.826	
3	Provide quality care through the best use of resources	0.812	
4	Contribute to the establishment of medication use policies, including anti-microbial stewardship, criteria, and maintenance of the formulary as a member of the Pharmacy and Therapeutics Committee	0.811	
5	Demonstrate an appropriate level of clinical knowledge related to medications and therapeutics in making decisions or recommendations related to the clinical use of drugs when appropriate	0.726	
Kaiser–Meyer–Olkin (KMO) 0.829; Bartlett’s test of sphericity < 0.001; Percentage of variance explained 65.05%			
Domain 3: Professional skills			
3.1. Written and Oral Communication			
1	Communicate with pharmacy and healthcare team members	0.881	0.844
2	Respond to questions with the appropriate level of detail necessary to ensure proper patient care and communication with other relevant parties	0.874	
3	Develop and maintain appropriate therapeutic recommendations related to medication therapy	0.833	
4	Demonstrate effective verbal and written communications	0.719	
Kaiser–Meyer–Olkin (KMO) 0.742; Bartlett’s test of sphericity < 0.001; Percentage of variance explained 68.72%			
3.2. Professional Advancement			
1	Engage in regular professional development activities	0.973	0.943
2	Engage in professional organization activities	0.973	
Kaiser–Meyer–Olkin (KMO) 0.500; Bartlett’s test of sphericity < 0.001; Percentage of variance explained 94.59%			
3.3. Behavior and Ethics			
1	Support staff in their professional and personal development	0.880	0.764
2	Able to carry out staff appraisals	0.855	
3	Apply ethical principles	0.720	
4	Demonstrate professional behavior (attitude, dress, appearance, etc.) in practice settings	0.620	
Kaiser–Meyer–Olkin (KMO) 0.681; Bartlett’s test of sphericity < 0.001; Percentage of variance explained 60.22%			
3.4. Management			
1	Prioritize multiple patient care and triage in times of high activity and workload	0.864	0.852
2	Implement the medication management strategy or plan	0.838	
3	Plan and manage physical and financial resources	0.829	
4	Demonstrate project and team management skills	0.728	
5	Demonstrate efficient problem-solving skills	0.719	
Kaiser–Meyer–Olkin (KMO) 0.755; Bartlett’s test of sphericity < 0.001; Percentage of variance explained 63.63%			
Domain 4: Pharmacist preparedness and response in emergency situation			
4.1. Emergency Preparedness and Response (EPR)			
1	Partner with local authorities	0.856	0.914
2	Balance stockpile and availability of drugs for existing/chronic conditions	0.851	
3	Address medication shortage and mitigation plan	0.806	
4	Follow actions and recommendations of local authorities	0.798	
5	Involve trainees and staff in emergency response	0.796	
6	Check for training opportunities	0.784	
7	Check for FDA/EMA Emergency Use Authorizations (EUAs) and expedited review and approval of tests/drugs for treatment	0.751	
8	Check for volunteering opportunities	0.712	
Kaiser–Meyer–Olkin (KMO) 0.879; Bartlett’s test of sphericity < 0.001; Percentage of variance explained 63.27%			
4.2. Operation management			
1	Adapt working hours to meet essential services during crises	0.888	0.918
2	Secure personal protective equipment or other needed materials	0.870	
3	Secure sanitizers and other medications when needed	0.857	
4	Develop workplace training and safety protocols (e.g., social distancing)	0.807	
5	Monitor workers/assistants for symptoms	0.798	
6	Participate in interdisciplinary training to emergency preparedness and response teams	0.790	
7	Ensure medication delivery/safe storage	0.719	
8	Procure essential medications and supplies	0.655	
Kaiser–Meyer–Olkin (KMO) 0.876; Bartlett’s test of sphericity < 0.001; Percentage of variance explained 64.23%			
4.3. Patient Care and Population Health Interventions			
1	Educate peers about the ongoing crisis using evidence-based information and communications	0.892	0.828
2	Identify at-risk populations	0.847	
3	Continue medication reviews, screening and/or testing/vaccination services safely	0.816	
4	Answer EPR-related calls	0.784	
5	Maintain patient confidentiality	0.483	
Kaiser–Meyer–Olkin (KMO) 0.792; Bartlett’s test of sphericity < 0.001; Percentage of variance explained 60.53%			
4.4. Evaluation, Research, and Dissemination for Impact and Outcomes			
1	Publish and/or disseminate findings	0.941	0.924
2	Participate in research and studies on EPR	0.919	
3	Develop training programs to peers and other healthcare workers	0.878	
4	Combat misinformation by disseminating evidence-based information to patients and sharing it on social media	0.870	
Kaiser–Meyer–Olkin (KMO) 0.831; Bartlett’s test of sphericity < 0.001; Percentage of variance explained 81.42%			

Table 3 displays clinical pharmacists’ competencies. Exploratory factor analysis shows the validity of the construct for the majority of competencies; patient data collection and assessment, interdisciplinary approach, professionalism and ethics, and leadership and self-management had very low variability of results. Moreover, the internal consistency of all competencies was shown to be high, except for leadership and self-management competencies, which showed a very low consistency value. Competencies were distributed on seven domains, i.e., quality improvement, clinical knowledge and skills, soft skills, ability to conduct clinical research, ability to provide effective education, use of information technology to make decisions, and emergency preparedness (Table 3).

**Table 3:** Factor analysis of Lebanese clinical pharmacists’ competencies (Promax rotated component matrix)

Competency		Loading	Cronbach alpha
Domain 0: Quality improvement			
0.1 Medication use management			
1	Understand the process for developing, implementing, and maintaining a formulary system	0.911	0.922
2	Implement quality improvement changes to the institution medication-use system	0.879	
3	Draft and distribute information and recommendations related to the clinical use of drugs when appropriate	0.811	
4	Participate in the identification of need for, development of, implementation of, and evaluation of an evidence-based treatment guideline/protocol related to individual and population-based patient care	0.811	
5	Participate in opportunities for improvement in the institutions medication-use system by comparing the medication-use system to relevant best practices	0.796	
6	Make a medication-use policy recommendation based on a comparative review, e.g., drug class review, drug monograph, antimicrobial stewardship, etc.	0.759	
7	Understand the institutions medication-use system and its vulnerabilities to adverse drug events (ADEs)	0.733	
8	Understand the structure and process of the medication-use system	0.706	
9	Understand the impact of pharmacist involvement on medication safety and quality using appropriate literature	0.706	
10	Document appropriate therapeutic recommendations related to medication therapy	0.679	
11	Use clinical pharmacy metrics/indicators to show the impact of clinical pharmacy services	0.660	
12	Identify opportunities for improvement of the institutions medication-use system	0.613	
13	Participate in pilot interventions to change problematic or potentially problematic aspects of the medication-use system with the objective of improving quality	0.562	
0.2 Medication dispensing			
1	Prepare and dispense medications following existing standards of practice and the institutions policies and procedures	0.934	0.900
2	Dispense medication products following the institutions policies and procedures	0.934	
3	Identify the appropriateness (rational and safe use) of a medication order before preparing or permitting the distribution of the first dose	0.868	
4	Prepare medication using appropriate techniques and following the institutions policies and procedures	0.868	
5	Follow the institutions policies and procedures to maintain the accuracy of the patients’ medication profile	0.694	
0.3 Workplace management			
1	Quality Improvement/Workplace Management [Understand the principles of financial management of a pharmacy department.]	0.961	0.890
2	Quality Improvement/Workplace Management [Use knowledge of the principles of change management to achieve organizational, departmental, and/or team goals.]	0.946	
3	Quality Improvement/Workplace Management [Evaluate the workload, organize the workflow, and check the accuracy of the work of pharmacy staff or others.]	0.827	
4	Quality Improvement/Workplace Management [Understand the effect of accreditation, legal, regulatory, and safety requirements on practice.]	0.825	
Domain 1: Clinical Knowledge and Skills			
1.0. Analytical Skills			
1	Provide concise, applicable, comprehensive, and timely responses to requests for drug information from patients and healthcare providers	0.949	0.909
2	Evaluate the usefulness of biomedical literature gathered	0.949	
3	Assess the effectiveness of drug information recommendations	0.879	
4	Determine relevant information to evaluate from all retrieved biomedical literature	0.879	
5	Formulate a systematic, efficient, and thorough procedure for retrieving drug information	0.843	
6	Formulate responses to drug information requests based on analysis of the literature	0.635	
7	Provide appropriate responses to drug information questions that require pharmacists to draw upon their knowledge base	0.422	
1.1. Fundamentals of Clinical Knowledge			
1	Support the management of acute toxicity and advice on appropriate antidotes	0.940	0.845
2	Apply knowledge of pathophysiology to specific therapeutic areas and to particular patient groups	0.929	
3	Discuss the importance of emerging technologies in pharmacology and pharmacotherapy	0.847	
4	Understand the scientific basis of different dosage formulations, and adjuvant compounds, their design and influence on the clinical efficacy of medicines	0.814	
5	Know the advantages and risks of the new formulations	0.801	
6	Understand how administration, drug distribution, drug elimination influences medicines outcomes	0.686	
7	Describe and discuss the pharmacology and pharmacotherapy of drugs in routine use	0.582	
8	Show knowledge of basic physical health examinations and laboratory tests and show ability to interpret and respond adequately to such data	0.458	
9	Understand normal organ anatomy and function, the effects of disease states that affect medicines use	0.457	
10	Demonstrate an appropriate level of clinical knowledge related to medications and therapeutics in making decisions or recommendations	0.381	
11	Identify pharmacotherapy-induced resistance and anti-microbial stewardship	0.125	
1.2. Patient Data Collection, Assessment, and Therapeutic Planning Skills			
1	Collect and organize all patient-specific information needed by the pharmacist to prevent, detect, and resolve medication-related problems and make appropriate evidence-based, patient-centered medication therapy recommendations as part of the interdisciplinary team	1.000	0.768
2	Determine if the medication is used with no medical indication	1.000	
3	Determine if the immunization regimen is incomplete	1.000	
4	Determine if the current medication therapy regimen contains something inappropriate (dose, dosage form, duration, schedule, route of administration, method of administration)	1.000	
5	Determine if there is therapeutic duplication	1.000	
6	Determine if the medication to which the patient is allergic has been prescribed	1.000	
7	Determine the presence of adverse drug or device-related events or potential for such events	1.000	
8	Determine clinically significant drug–drug, drug–disease, drug–nutrient, or drug–laboratory test interactions or potential for such interactions	1.000	
9	Determine if the medical therapy has been interfered with by social, recreational, non-prescription, or non-traditional drug use by the patient	1.000	
10	Determine if the patient is not receiving the full benefit of prescribed medication therapy	1.000	
11	Determine if there are problems arising from financial impact of medication therapy on the patient	1.000	
12	Determine if the patient lacks understanding of medication therapy	1.000	
13	Determine if the patient is not adhering to medication regimen	1.000	
14	Assess options available for problem solving, considering possible outcomes of therapeutic actions	1.000	
15	Use benefit–risk assessments for evaluating alternative treatment strategies	1.000	
16	Optimize use of drugs including: addition, deletion, dose adjustment, IV to Po switch, renal dosing, dose reduction, etc.	1.000	
17	Use an organized collection of patient-specific information, summarize patients’ healthcare needs	1.000	
18	Evaluate medication-use patterns in a specified patient population (geriatrics, pediatrics, etc.)	1.000	
19	Make a referral to the appropriate healthcare provider based on the patient’s acuity and the presenting problem, when presented with a patient with healthcare needs that cannot be met by the pharmacist	1.000	
20	Design an evidence-based therapeutic regimen	1.000	
21	Identify therapeutic goals and design a patient-centered regimen that meets the evidence-based therapeutic goals established for a patient	1.000	
22	Integrate patient-specific information, disease and drug information, ethical and quality of life issues	1.000	
23	Consider pharmacoeconomic principles: identify cost effective medicines and medical devices using valid and relevant pharmacoeconomic data	1.000	
24	Note: Unidimensional; full loading; low variance		
1.3. Monitoring and Follow-Up Skills			
1	Accurately assess the patient’s progress toward the therapeutic goal(s)	0.893	0.865
2	Redesign a patient-centered, evidence-based therapeutic plan as necessary based on the evaluation of monitoring data and therapeutic outcomes	0.882	
3	When appropriate, initiate the patient-centered, evidence-based therapeutic regimen and monitoring plan for a patient according to the institutions’ policies and procedures	0.882	
4	Design a patient-centered, evidence-based monitoring plan for a therapeutic regimen that effectively evaluates the achievement of patient-specific goals	0.720	
5	Kaiser–Meyer–Olkin (KMO) 0.412, Bartlett’s test of sphericity < 0.001, Percentage of variance explained 71.78%		
1.4. Medication Safety and Surveillance			
1	Consider that reporting an ADR is part of pharmacist duties	0.932	0.565
2	Identify high-risk medicines and high-risk administration of medicines relevant to the healthcare setting	0.932	
3	Plan and implement medicines management actions to minimize the risk related to these medicines	0.852	
4	Identify a potential adverse drug reaction	0.663	
5	Demonstrate knowledge on reporting an adverse drug reaction to relevant authorities	0.174	
1.5. Transition of Care and Reconciliation Skills			
1	Document, reconcile and update patient medication history and received interventions	0.434	0.197
2	Communicate pertinent pharmacotherapeutic information to the receiving healthcare professionals, when a patient is transitioning from one healthcare setting to another	0.434	
3	Ensure that accurate and timely medication-specific information reconciliation procedure regarding a specific patient reaches those who need it at the appropriate time	0.911	
4	Note: Unidimensional; full loading; low variance		
Domain 2: soft skills			
2.0. Communication skills			
1	Use effective communication practices when documenting a direct patient-care activity	0.858	0.518
2	Use effective patient education techniques to provide counseling to patients and caregivers, including information on medication therapy, adverse effects, compliance, appropriate use, handling, and medication administration	0.799	
3	Provide clear and concise consultations to other health professionals	0.788	
4	Explain the characteristics of exemplary documentation systems that may be used in the organizations’ environment	− 0.263	
5	Appropriately select direct patient-care activities for documentation	0.201	
2.2. Interdisciplinary approach			
1	Demonstrate cooperative, collaborative, and communicative working relationships with members of interdisciplinary healthcare teams as appropriate	1.000	0.794
2	Prioritize and manage daily activities to deliver appropriate patient-centered care to each patient	1.000	
3	Demonstrate collaborative professional pharmacist–patient relationships as appropriate	1.000	
4	Recommend or communicate a patient-centered, evidence-based therapeutic regimen and corresponding monitoring plan to other members of the interdisciplinary team and patients systematically, logically, accurately, and timely and secure consensus from the team and patient	1.000	
Unidimensional; full loading; low variance			
2.3. Professionalism, ethics, and patient advocacy			
1	Demonstrate pride in and commitment to the profession through appearance, personal conduct, and association membership	1.000	0.774
2	Act ethically in the conduct of all job-related activities	1.000	
3	Demonstrate ownership of and responsibility for the welfare of the patient by addressing pharmacy-related patient care problems	1.000	
4	Respect the rights of patients in therapeutic decisions and assist in providing information to facilitate their decision	1.000	
5	Respect and maintain the individuals’ right to confidentiality	1.000	
6	Engage in regular professional development activities	1.000	
7	Engage in professional organization activities	1.000	
Unidimensional; full loading; low variance			
2.4. Leadership and Self-Management			
1	Demonstrate pride in and commitment to the profession through appearance, personal conduct, and association membership	1.000	0.284
2	Act ethically in the conduct of all job-related activities	1.000	
3	Demonstrate ownership of and responsibility for the welfare of the patient by addressing pharmacy-related patient care problems	1.000	
4	Respect the rights of patients in therapeutic decisions and assist in providing information to facilitate their decision	1.000	
5	Respect and maintain the individuals’ right to confidentiality	1.000	
Unidimensional; full loading; low variance			
Domain 3: Ability to conduct clinical research			
3.1. Research Project Management Skills			
1	Employ accepted manuscript style to prepare a final report of a practice-related project	0.969	0.957
2	Implement a practice-related project as specified in its design (clinical trials and observational studies)	0.919	
3	Describe, explain and discuss commonly used research methodologies	0.897	
4	Suggest a feasible design for a practice-related project	0.891	
5	Effectively present the results of a practice-related project	0.868	
6	Secure any necessary approvals, including IRB, for one’s design of a practice-related project	0.861	
7	Participate in pharmacy practice research and clinical research	0.850	
Domain 4: Ability to provide effective education			
4.1. Educational skills			
1	Use effective educational techniques in the design of all educational activities	0.876	0.397
2	Use knowledge of audio–visual aids and handouts to enhance the effectiveness of communications	0.550	
3	Use public speaking skills to speak effectively in large and small group situations	0.476	
4	Use skill in case-based teaching	0.476	
Domain 5: Use information technology to make decisions and reduce errors			
5.1. Informatics Skills			
1	Exercise skill in elementary use of databases and data analysis software	0.952	0.815
2	Explain security and patient protections such as access control, data security, data encryption, privacy regulations, as well as ethical and legal issues related to the use of information technology in pharmacy practice	0.866	
3	Use healthcare delivery systems and health informatics to optimize the care of individual patients and patient populations	0.761	
Domain 6: Pharmacist preparedness and response in emergency situations			
6.1. Emergency Preparedness and Response (EPR)			
1	Follow actions and recommendations of local authorities	0.983	0.977
2	Address medication shortage and mitigation plan	0.963	
3	Balance stockpile and availability of drugs for existing/chronic conditions	0.963	
4	Check for FDA/EMA Emergency Use Authorizations (EUAs) and expedited review and approval of tests/drugs for treatment	0.961	
5	Check for training opportunities	0.926	
6	Partner with local authorities	0.905	
7	Check for volunteering opportunities	0.875	
6.2. Operation management			
1	Secure PPEs or other needed materials	0.982	0.988
2	Participate in interdisciplinary training to EPR teams	0.976	
3	Procure essential medications and supplies	0.972	
4	Develop workplace training and safety protocols (e.g., social distancing)	0.968	
5	Monitor workers/assistants for symptoms	0.968	
6	Secure sanitizers and other medications when needed	0.967	
7	Ensure medication delivery/safe storage	0.936	
8	Adapt working hours to meet essential services during crises	0.919	
6.3. Patient care and population health interventions			
1	Manage panic buying	0.968	0.877
2	Answer EPR-related calls	0.966	
3	Identify at-risk populations	0.965	
4	Educate patient about the ongoing crisis using evidence-based information and communications	0.952	
5	Continue medication reviews, screening and/or testing/vaccination services safely	0.146	
6	Maintain patient confidentiality	0.114	
6.4. Evaluation, research, and dissemination for impact and outcomes			
1	Participate in research and studies on EPR	0.940	0.879
2	Publish and/or disseminate findings	0.940	
3	Combat misinformation by disseminating evidence-based information to patients and sharing it on social media	0.934	
4	Develop training programs to peers and other healthcare workers	0.711	

Tables 4 and 5 describe hospital and clinical pharmacy competencies domains. Pharmacists were highly confident in most competencies, with some exceptions related to research in emergency settings (data evaluation, research, and reporting).

**Table 4:** Description of hospital pharmacists’ competency domains

	Mean ± SD	% of mean	Median	Minimum	Maximum
Domain 0: fundamental skills	157.37 ± 16.97	87.42%	158.00	109.00	180.00
0.1 Regulations	23.16 ± 2.44	92.64%	24.00	10.00	25.00
0.2 Drug procurement and management	40.32 ± 4.83	89.60%	41.00	21.00	45.00
0.3 Optimize processes related to organization, medication preparation, and delivery	14.19 ± 1.18	94.60%	15.00	10.00	15.00
0.4 Aseptic techniques	13.41 ± 2.09	89.40%	14.00	6.00	15.00
0.5 Pharmaceutical and hospital technology/automation	12.34 ± 2.88	82.26%	13.00	3.00	15.00
0.6 Pharmaceutical/medical skills	37.73 ± 6.52	83.84%	38.00	19.00	45.00
0.7. Business management skills	16.20 ± 3.91	81.00%	17.00	4.00	20.00
Domain 1: safe and rational use of medicines	64.05 ± 6.29	91.50%	67.00	46.00	70.00
1.1 Patient safety	31.96 ± 3.57	91.31%	34.00	20.00	35.00
1.2 Quality assurance	18.58 ± 1.83	92.90%	20.00	13.00	20.00
1.3 Pharmacovigilance	13.50 ± 1.75	90.00%	14.00	8.00	15.00
Domain 2: patient-centered care	56.78 ± 8.09	87.35%	59.00	32.00	65.00
2.1 Literature evaluation/search/trials	17.35 ± 2.61	86.75%	18.00	8.00	20.00
2.2. Pharmacokinetic-based assessment	16.68 ± 3.68	83.40%	18.00	7.00	20.00
2.3. Drug use optimization	22.73 ± 2.64	90.92%	24.00	15.00	25.00
Domain 3: professional skills	68.91 ± 5.94	91.88%	71.00	52.00	75.00
3.1 Written and oral communication	18.91 ± 1.59	94.55%	20.00	14.00	20.00
3.2 Professional advancement	8.90 ± 1.35	89.00%	10.00	4.00	10.00
3.3 Behavior and ethics	18.85 ± 1.63	94.25%	20.00	14.00	20.00
3.4 Management	22.24 ± 2.81	88.96%	23.00	14.00	25.00
Domain 4: pharmacist preparedness and response in emergency situation	108.17 ± 13.53	86.53%	109.00	74.00	125.00
4.1 Emergency preparedness and response (EPR)	34.14 ± 5.15	85.35%	35.00	18.00	40.00
4.2. Operation management	36.27 ± 3.90	90.67%	37.00	24.00	40.00
4.3. Patient care and population health interventions	22.25 ± 2.78	89.00%	22.00	13.00	25.00
4.4. Evaluation, research, and dissemination for impact and outcomes	15.49 ± 3.86	77.45%	16.00	5.00	20.00

**Table 5:** Description of clinical pharmacists’ competency domains

	Mean ± SD	% of mean	Median	Minimum	Maximum
Domain 0: quality improvement	99.40 ± 11.33	90.36%	101.50	72.00	110.00
0.1 Medication use management	58.30 ± 6.83	89.69%	59.50	44.00	65.00
0.2 Medication Dispensing	24.20 ± 1.61	96.80%	25.00	20.00	25.00
0.3 Workplace management	16.90 ± 3.81	84.50%	18.00	8.00	20.00
Domain 1: clinical knowledge and skills	249.40 ± 10.83	94.11%	247.50	232.00	265.00
1.1 Analytical skills	32.90 ± 2.64	94.00%	34.00	28.00	35.00
1.2 Fundamentals of clinical knowledge	49.60 ± 4.85	90.18%	50.00	38.00	55.00
1.3 Patient data collection, assessment, and therapeutic planning skills	110.40 ± 3.89	96.00%	111.00	103.00	115.00
1.4. Monitoring and follow-up skills	18.50 ± 1.71	92.50%	19.00	16.00	20.00
1.5. Transition of care and reconciliation skills	14.30 ± 0.82	95.33%	14.50	13.00	15.00
1.6. Medication safety and surveillance	23.70 ± 1.70	94.80%	24.50	20.00	25.00
Domain 2: soft skills	102.00 ± 3.80	97.14%	103.50	94.00	105.00
2.1 Communication Skills	23.60 ± 1.34	94.40%	24.00	21.00	25.00
2.2 Interdisciplinary approach	19.60 ± 0.96	98.00%	20.00	17.00	20.00
2.3. Professionalism, Ethics, and Patient Advocacy	34.20 ± 1.47	97.71%	35.00	31.00	35.00
2.4. Leadership and Self-Management	24.60 ± 0.69	98.40%	25.00	23.00	25.00
Domain 3: ability to conduct clinical research					
3.1 Research Project Management Skills	30.70 ± 4.29	87.71%	31.00	24.00	35.00
Domain 4: ability to provide effective education					
4.1 Educational Skills	18.70 ± 1.15	93.50%	19.00	16.00	20.00
Domain 5: use information technology to make decisions and reduce errors					
5.1. Informatics Skills	12.70 ± 2.75	84.66%	14.00	8.00	15.00
Domain 6: pharmacist preparedness and response in emergency situations	103.90 ± 23.98	83.12%	111.00	49.00	125.00
6.1. Emergency Preparedness and Response (EPR)	28.60 ± 9.08	81.71%	32.50	7.00	35.00
6.2. Operation Management	32.70 ± 10.54	81.75%	36.50	8.00	40.00
6.3. Patient Care and Population Health Interventions	26.50 ± 4.90	88.33%	28.50	14.00	30.00
6.4. Evaluation, Research, and Dissemination for Impact and Outcomes	16.10 ± 2.96	80.50%	15.50	12.00	20.00

In the bivariate analysis, hospital pharmacists who had higher degrees than a BS Pharm were more confident in Domain 3 (professional skills); those who worked in larger hospitals were more confident in Domains 1 and 3 (safe and rational use of medications and professional skills), and those who had other fields of work were more confident in Domain 1 (safe and rational use of medications) (Table 6).

**Table 6:** Bivariate analysis taking hospital pharmacists’ competency domains as the dependent variables

		Domain 0 Fundamental Skills	Domain 1 Safe and Rational Use of Medicines	Domain 2 Patient-centered care	Domain 3 Professional Skills	Domain 4 Emergency preparedness and response
Gender						
Male		154.07 ± 18.77	64.57 ± 6.93	57.28 ± 7.22	68.50 ± 7.24	108.64 ± 15.52
Female		158.01 ± 16.67	63.95 ± 6.21	56.68 ± 8.28	69.00 ± 5.72	108.08 ± 13.24
* p* value		0.429	0.741	0.801	0.775	0.888
Level of education						
BS Pharmacy	Yes	157.39 ± 17.44	64.12 ± 6.07	56.81 ± 8.08	68.92 ± 6.00	108.22 ± 13.80
	No	157.25 ± 12.20	63.37 ± 8.66	56.50 ± 8.68	68.87 ± 5.74	107.62 ± 11.23
	*p* value	0.982	0.817	0.918	0.982	0.905
PharmD	Yes	155.10 ± 19.44	63.70 ± 6.57	55.93 ± 9.15	68.53 ± 6.17	107.23 ± 14.63
	No	160.05 ± 13.27	64.47 ± 6.02	57.77 ± 6.61	69.37 ± 5.71	109.27 ± 12.20
	*p* value	0.165	0.571	0.281	0.513	0.487
Masters	Yes	155.56 ± 21.52	65.34 ± 5.24	56.91 ± 8.09	70.17 ± 6.11	109.26 ± 13.14
	No	158.03 ± 15.16	63.59 ± 6.61	56.73 ± 8.15	68.46 ± 5.86	107.78 ± 13.75
	*p* value	0.553	0.254	0.928	0.241	0.656
PhD	Yes	166.00 ± 7.07	67.50 ± 0.70	63.00 ± 0.001	69.50 ± 7.77	117.00 ± 5.65
	No	157.17 ± 17.10	63.97 ± 6.35	56.63 ± 8.12	68.90 ± 5.95	107.96 ± 13.61
	*p* value	0.471	0.437	0.274	0.890	0.354
Highest degree related to your main field of work						
BS/PharmD/DU		156.66 ± 16.88	63.55 ± 6.40	56.24 ± 8.50	68.15 ± 5.95	107.46 ± 14.04
Master/PhD/Postgraduate		160.11 ± 17.53	66.00 ± 5.62	58.83 ± 6.03	71.83 ± 5.09	110.88 ± 11.32
* p* value		0.447	0.143	0.229	0.019	0.342
Language of pharmacy education						
French		157.33 ± 20.18	63.36 ± 7.21	54.86 ± 9.02	68.83 ± 6.38	106.23 ± 14.82
English		157.00 ± 15.04	64.32 ± 5.79	57.66 ± 7.44	68.91 ± 5.80	108.92 ± 12.78
* p* value		0.931	0.535	0.153	0.955	0.381
University of graduation as a pharmacist						
UL		154.95 ± 21.05	62.62 ± 7.14	53.95 ± 10.35	67.58 ± 7.08	104.54 ± 16.16
LIU		160.54 ± 13.25	64.20 ± 6.57	57.87 ± 6.50	68.66 ± 5.98	109.83 ± 10.90
Other		156.92 ± 16.35	64.84 ± 5.54	57.84 ± 7.12	69.89 ± 5.08	109.38 ± 13.15
* p* value		0.515	0.398	0.133	0.319	0.305
University of the highest earned degree						
UL		154.95 ± 18.91	63.12 ± 6.36	54.04 ± 9.59	68.08 ± 6.54	105.54 ± 14.39
LIU		160.72 ± 13.83	63.90 ± 6.79	57.72 ± 6.69	68.59 ± 6.02	109.54 ± 11.36
Other		157.00 ± 17.42	64.68 ± 6.07	57.87 ± 7.61	69.58 ± 5.60	108.97 ± 14.18
* p* value		0.511	0.630	0.149	0.595	0.533
Work Location						
Beirut		158.17 ± 16.59	64.91 ± 5.93	58.23 ± 6.51	69.14 ± 6.32	106.67 ± 13.52
Mount Lebanon		158.42 ± 14.71	65.80 ± 4.74	57.80 ± 8.55	70.28 ± 5.28	112.47 ± 12.46
South		155.08 ± 20.81	63.33 ± 7.48	56.41 ± 8.18	68.58 ± 6.55	108.41 ± 14.49
North		154.27 ± 22.13	61.45 ± 6.23	53.54 ± 10.47	66.81 ± 6.19	106.00 ± 16.25
Beqaa		158.77 ± 13.58	60.88 ± 8.16	53.33 ± 8.76	67.88 ± 5.08	106.11 ± 11.77
* p* value		0.941	0.159	0.306	0.594	0.561
Number of beds at the hospital						
< 50		155.11 ± 16.31	64.61 ± 4.96	56.38 ± 8.28	69.16 ± 5.23	109.33 ± 10.05
50–100		156.22 ± 17.99	61.61 ± 7.93	54.45 ± 8.59	66.64 ± 7.17	104.45 ± 15.87
101–300		157.20 ± 17.92	65.62 ± 4.97	58.86 ± 7.58	70.31 ± 4.98	109.86 ± 13.64
> 300		166.44 ± 9.20	66.33 ± 3.64	58.88 ± 6.15	71.77 ± 2.38	113.22 ± 7.96
* p* value		0.392	**0.048**	0.161	**0.038**	0.245
Having another field of work						
I do not have another field of work		156.00 ± 17.68	62.62 ± 7.00	55.74 ± 8.02	68.52 ± 6.29	107.80 ± 13.79
I have another work		159.33 ± 15.96	66.08 ± 4.50	58.25 ± 8.06	69.47 ± 5.45	108.69 ± 13.34
* p* value		0.370	**0.006**	0.156	0.470	0.764

	Correlation coefficient	Correlation coefficient	Correlation coefficient	Correlation coefficient	Correlation coefficient
Age	− 0.002	− 0.012	− 0.207	0.022	− 0.015
*p* value	0.989	0.912	0.054	0.841	0.892
Number of working hours per day	0.015	0.124	0.061	0.089	0.031
*p* value	0.891	0.251	0.573	0.411	0.776
Number of working days per week	− 0.092	0.0001	− 0.071	− 0.119	− 0.032
*p* value	0.398	0.998	0.511	.272	0.769
Years of experience	0.129	0.043	-0.092	0.082	0.025
*p* value	0.232	0.695	0.398	0.452	0.820

*LIU* Lebanese International University, *UL* Lebanese University

Clinical pharmacists, who were English educated, had a BS Pharm, and graduated from LAU were more confident in Domain 3 (soft skills) (Table 7).

**Table 7:** Bivariate analysis taking clinical pharmacists’ competency domains as the dependent variables

		Domain 0 Quality Improvement	Domain 1 Clinical Knowledge and Skills	Domain 2 Soft skills	Domain 3 Ability to Conduct Clinical Research	Domain 4 Ability to Provide Effective Education	Domain 5 Use Information Technology to Make Decisions and Reduce Errors	Domain 6 Emergency preparedness and response
Gender								
Male		104.66 ± 8.38	251.00 ± 12.28	98.66 ± 5.68	31.33 ± 3.51	19.00 ± 1.00	12.66 ± 3.21	114.66 ± 9.29
Female		97.14 ± 12.22	248.71 ± 11.13	103.42 ± 1.71	30.42 ± 4.82	18.57 ± 1.27	12.71 ± 2.81	99.28 ± 27.40
* p* value		0.366	0.779	0.282	0.780	0.622	0.982	0.384
Level of education								
BS Pharmacy	Yes	98.57 ± 13.26	248.00 ± 11.87	102.14 ± 3.97	32.42 ± 3.77	18.57 ± 1.39	12.71 ± 2.81	99.28 ± 27.62
	No	101.33 ± 6.50	252.66 ± 9.07	101.66 ± 4.16	26.66 ± 2.30	19.00 ± 0.001	12.66 ± 3.21	114.66 ± 7.09
	*p* value	0.746	0.564	0.868	0.043	0.622	0.982	0.384
Master’s degree	Yes	103.85 ± 6.25	251.57 ± 10.90	101.14 ± 4.29	29.85 ± 4.41	18.42 ± 1.27	13.57 ± 2.29	113.42 ± 8.16
	No	89.00 ± 15.13	244.33 ± 10.78	104.00 ± 1.00	32.66 ± 4.04	19.33 ± 0.57	10.66 ± 3.05	81.66 ± 6.46
	*p* value	0.226	0.363	0.140	0.374	0.283	0.132	0.269
Highest degree related to your main field of work								
PharmD		92.75 ± 14.08	241.25 ± 7.18	102.50 ± 1.73	29.75 ± 4.27	18.00 ± 1.41	12.25 ± 3.09	102.00 ± 19.61
Master’s		105.80 ± 6.14	255.40 ± 10.59	101.00 ± 5.14	30.60 ± 4.72	19.00 ± 0.70	13.60 ± 2.60	116.40 ± 7.30
*p* value		0.101	0.057	0.568	0.788	0.206	0.500	0.168
Language of pharmacy education								
French		92.25 ± 14.66	251.50 ± 9.97	100.66 ± 4.50	28.50 ± 4.03	18.83 ± 0.75	13.66 ± 2.33	115.16 ± 6.85
English		104.16 ± 5.84	246.25 ± 12.81	104.00 ± 0.81	34.00 ± 2.00	18.50 ± 1.73	11.25 ± 2.98	87.00 ± 1.82
* p* value		0.105	0.486	0.132	0.037	0.682	0.188	0.174
University of the highest earned degree								
USJ		103.40 ± 6.18	249.20 ± 9.20	99.80 ± 4.43	29.40 ± 3.78	18.80 ± 0.83	13.40 ± 2.50	115.00 ± 7.64
Other		95.40 ± 14.51	249.60 ± 13.39	104.20 ± 0.83	32.00 ± 4.79	18.60 ± 1.51	12.00 ± 3.08	92.80 ± 30.45
* p* value		0.290	0.957	0.090	0.369	0.803	0.454	0.181
Work Location								
Beirut		98.50 ± 12.10	248.62 ± 10.14	101.50 ± 4.10	30.12 ± 4.54	18.87 ± 0.64	12.50 ± 2.97	101.50 ± 25.86
Mount Lebanon		103.00 ± 9.89	252.50 ± 17.67	104.00 ± 1.41	33.00 ± 2.82	18.00 ± 2.82	13.50 ± 2.12	113.50 ± 16.26
* p* value		0.644	0.678	0.438	0.430	0.737	0.672	0.559
Providing direct patient care								
On rounds		97.16 ± 13.94	245.83 ± 11.39	101.83 ± 4.26	32.00 ± 3.94	18.50 ± 1.51	12.33 ± 2.87	95.50 ± 28.20
On rounds and telehealth		102.75 ± 6.02	254.75 ± 8.50	102.25 ± 3.59	28.75 ± 4.57	19.00 ± 0.01	13.25 ± 2.87	116.50 ± 6.85
* p* value		0.478	0.221	0.877	0.264	0.456	0.635	0.132
Having another field of work								
I do not have another field of work		102.16 ± 6.30	247.66 ± 9.04	100.33 ± 4.17	29.66 ± 3.44	18.33 ± 1.36	13.16 ± 2.31	112.83 ± 8.65
I have another work		95.25 ± 16.76	252.00 ± 14.16	104.50 ± 0.57	32.25 ± 5.50	19.25 ± 0.50	12.00 ± 3.55	90.50 ± 34.66
* p* value		0.375	0.567	0.058	0.383	0.242	0.543	0.290

	Correlation coefficient	Correlation coefficient	Correlation coefficient	Correlation coefficient	Correlation coefficient	Correlation coefficient	Correlation coefficient
Age	0.084	0.516	0.511	− 0.002	0.466	− 0.030	− 0.453
*p* value	0.817	0.127	0.131	0.996	0.174	0.935	0.188
Number of working hours per day	− 0.146	0.023	− 0.084	− 0.266	0.247	− 0.300	− 0.321
*p* value	0.688	0.949	0.818	0.457	0.492	0.399	0.366
Number of working days per week	− 0.410	− 0.238	0.363	0.477	− 0.218	− 0.259	− 0.093
*p* value	0.239	0.508	0.302	0.164	0.545	0.470	0.798
Years of experience	0.287	0.650	0.471	0.053	0.503	0.148	− 0.322
*p* value	0.421	0.042	0.169	0.885	0.138	0.683	0.364

*LAU* Lebanese American University, *USJ* Saint Joseph University of Beirut

## Discussion

This study could validate competency frameworks for clinical and hospital pharmacists. The factor analysis of the competencies and their respective behaviors showed an adequate construct analysis. Moreover, Cronbach alpha values were appropriate, indicating sufficient to high internal consistency.

Overall, the competencies were distributed over five domains for hospital pharmacists, i.e., fundamental skills, safe and rational use of medicines, patient-centered care, professional skills, and emergency preparedness. From the content point of view, these domains are similar (although not parallel) to those suggested by the European Association of Hospital Pharmacists [33, 34] (i.e., patient care, medicines and their use, management competencies, and professional competencies), except for the emergency preparedness aspect that was added in the current framework. Similarly, at the global level, the Basel statements (2008) included standards (not competency domains); they covered all areas of medication management in a hospital setting, including procurement, preparation and delivery, prescribing, administration monitoring of patient outcomes, and human resources, but not emergency preparedness aspects. The standards are to be upgraded in 2024 by the FIP [35, 36]. Mapping this framework to the new statements would be of primary interest.

For clinical pharmacists, competencies were distributed over seven domains, i.e., quality improvement, clinical knowledge and skills, soft skills, ability to conduct clinical research, ability to provide effective education, use information technology to make decisions and reduce errors, and emergency preparedness. The domains derived from this study encompass those suggested by the American College of Clinical Pharmacy [4] (direct patient care, pharmacotherapy knowledge, system-based care and population health, communication, professionalism, and continuous professional development) and add the areas of research, education, information technology, and emergency preparedness. However, except for emergency preparedness, the competency domains suggested in this study are closer to those developed in Sweden [37], which include clinical pharmacy practice, working relationships and communication, leadership and motivation, service development, education and training, and research and evaluation. Further mapping exercises and validation studies are warranted to confirm the validity of the suggested framework.

As for assessment results, hospital pharmacists seemed to have had more years of work experience than clinical pharmacists, most probably because of the lack of regulations requiring the presence of clinical pharmacists in hospitals throughout Lebanon [38, 39]. Although patient-centered care was provided by both hospital and clinical pharmacists, as evidenced by Domains 1 and 2 in hospital pharmacists and Domain 1 in clinical pharmacists, the reported confidence in these domains varied, being higher among clinical pharmacists. Pharmacists working in hospital settings in Lebanon, including clinical and hospital pharmacists, reported receiving appropriate orientation into their career paths from their universities. Despite adequate guidance, the professional satisfaction of having their knowledge and skills fully used was very low at 18% in comparison with other career paths [38]. Not only does this finding emphasize the importance of advocating for pharmacy practice reform as previously suggested [40], but it also calls for pharmacists and pharmacy leaders to exhibit more leadership in advocating for the full utilization of pharmacists’ skills and competencies in hospital settings. Pharmacists could be offered continuing education sessions that tackle this particular competency to enhance their expertise and satisfaction.

Indeed, the exploratory factor analysis demonstrated the validity of the construct for the majority of the competencies, except for leadership and self-management, which showed a considerably low consistency value and might be a sign of differential confidence in various leadership aspects. Several studies have identified the lack of leadership among pharmacy students in the United States [41–43]. In Lebanon, in a previous study, LAU pharmacy students reported that the curriculum provided them with more theoretical than practical opportunities to develop their leadership skills [44]. Nevertheless, a recent study conducted in Lebanon [45] showed that 60–70% of pharmacists reported appropriate leadership and management behaviors, but pharmacists’ overconfidence in their competencies could not be ruled out. Further studies on pharmacists’ leadership are required to depict this finding, particularly in the hospital setting. In parallel, many pharmacy schools in the United States have developed new leadership courses, programs, retreats, or other extracurricular activities in response to the recognition of leadership by different pharmacy organizations and accrediting bodies [46–49]. In Lebanon, efforts are still needed at the undergraduate and continuing education levels to improve pharmacists’ leadership.

As per Lebanese law, pharmacists are permitted to teach even if they work full-time in other institutions [38]. Both hospital and clinical pharmacists were involved in teaching/precepting 17.2% and 40%, respectively. Although the current study did not distinguish between pharmacists teaching on or off hospital premises, if it is on hospital premises, such arrangements are beneficial to all parties involved, pharmacists, student pharmacists, institutions, and patients. Student pharmacists are given the opportunity to implement the skills learned in didactic and laboratory courses, such as the pharmacist patient care process, and contribute to error interception [11, 50]. Pharmacists and institutions also benefit from having student learners as pharmacist extenders, bridging the gap from academia to practice [51].

Pharmacists who worked in larger hospitals were confident in the safe and rational use of medications and professional skills, and those who had additional fields of work were more confident in the safe and rational use of medications. This result was expected, since big hospitals are mainly teaching hospitals [39] with better clinical pharmacy services and better ranking on Lebanese accreditation systems that require continuing education for hospital staff [52]. Furthermore, pharmacists with additional fields of work might have more exposure to information, such as in academia. More studies are necessary to explore these findings.

In this study, clinical pharmacists who graduated from an American-system university had a higher confidence level in the soft skills domain. This finding should be interpreted cautiously due to the small sample size of clinical pharmacists. Nevertheless, it can be explained by the results of a study assessing the integration of the personal and professional development (PPD) subdomains (self-assessment, leadership, innovation and entrepreneurship, and professionalism) in the pharmacy curriculum at LAU School of Pharmacy. The four mapping activities performed found these subdomains to be woven across curricular, co-curricular, and extra-curricular activities and showed their sequential integration at different depths and breadths in the curriculum [44]. Such integration may be more evident in American universities, since it is required by accrediting bodies, such as the Accreditation Council for Pharmacy Education [53].

Pharmacists were highly confident in most competencies, with some exceptions related to research in emergency settings (data evaluation, research, and reporting). In 2021, the American Society of Health-System Pharmacists issued guidelines on Emergency Medicine Pharmacy Services, delineating the role of Emergency Medicine Pharmacists (EMPs), including emergency-based research and scholarly activities [54]. Pharmacy research in the emergency medicine environment is on the rise, exploring the impact of various clinical activities and describing the progress in the medication-use process and pharmacy activities in emergency settings [55]. In Lebanon, the role of hospital pharmacists in emergency settings is still undermined; an example being their role during the COVID-19 pandemic, where they had difficulties practicing despite their adequate knowledge [56]. Clinical and hospital pharmacy leaders should strive to provide the necessary evidence that demonstrates the benefit emergency medicine pharmacists can provide to the quality and safety of care in various emergency settings, such as pandemics, wars, and other disasters.

### Recommendations

A national assessment of domains and competencies related to soft skills and research in emergency settings is needed at multiple levels throughout the country, including academic curricula, postgraduate training programs, drafted legislation governing pharmacy practice, and hospital accreditation standards [57]. The development of observable tasks related to each competency domain would help provide feedback and identify areas needed for professional development [58].

It is essential to improve pharmacist competency domains related to leadership and self-management. It is also necessary to incorporate more leadership-focused educational opportunities into pharmacy education, such as curricular, co-curricular, and extracurricular activities for student pharmacists, and develop targeted research to assess leadership competency outcomes. Involving students and postgraduate trainees in interprofessional education and practice teaches leadership through a collaborative rather than a vertical hierarchical approach [59, 60]. The Order of Pharmacists of Lebanon is also encouraged to establish training programs and provide continuing education opportunities that promote leadership among pharmacists [61]. Finally, advocacy for the pharmacy profession can help improve pharmacist job satisfaction and, in turn, stimulate interest, opportunities, and competency development in leadership [45].

Interventions can be instituted at multiple levels across the country to develop pharmacist competencies related to research in emergency settings. Given the recent disaster experiences in Lebanon, i.e., the COVID-19 pandemic and the Beirut blast on August 4, 2020, creating a repository of accessible publications related to pharmacist interventions or interprofessional interventions and improved patient outcomes would provide recognition for the role of pharmacists in emergency preparedness and response [29]. Moreover, such databases would serve as resources for competency development in this domain. At the academic level, pharmacy curricula across the country could educate students about emergency preparedness and response and collaborate to develop joint research initiatives to strengthen this competency among graduating professionals [40].

### Limitations

Despite its importance, this pilot study has several limitations to consider. A possible selection bias could exist due to the limited number of participants in the assessment. However, even with larger samples, the reliable structure of the framework and the significant associations and differences are expected to remain unchanged. Another possible limitation is information bias, which may arise due to the lengthy questionnaire and potential fatigue of participants, in addition to the self-reported nature of the information and the possibility of not understanding all questions. Indeed, no hospital pharmacists answered positively on the direct patient care question, although they are known to review medication prescriptions in most of the Lebanese hospitals [39], as recommended by the Lebanese accreditation standards for hospitals [62]; in addition, hospital pharmacists holding degrees higher than a BS Pharm were more confident in the professional skills domain than holders of higher education degrees, which is probably an overestimation of their own performance. Nevertheless, the suspected information bias would be non-differential and only lead to results leaning toward the null. Finally, confounding could not be reduced through multivariable analysis during the assessment of associations. Therefore, further large-scale studies are recommended to overcome these limitations and confirm our results.

## Conclusion

This study could validate competency frameworks for clinical and hospital pharmacists, with the competencies and their respective behaviors showing an adequate construct analysis. It also examined hospital and clinical pharmacists’ perceptions of the domains that need strengthening for an optimal public health system. Notably, domains that require further development are the soft skill domains and research in emergency settings. Both these domains are timely and needed to overcome the current practice challenges in Lebanon. A national assessment of these domains and competencies is needed at multiple levels to help develop observable tasks that would provide feedback and identify areas needed for professional development.

## Supplementary Information

**Additional file 1.** Advanced Competencies for Hospital Pharmacists.

**Additional file 2.** Advanced Competencies for Clinical Pharmacists.

## Data Availability

The data sets generated during and/or analyzed during the current study are available from the corresponding author upon reasonable request.
