# Supplementary material for: Validation of the specialized competency framework for pharmacists in hospital settings (SCF–PHS): a cross-sectional study
Source: J Pharm Policy Pract. 2023 Jul 10;16:86. doi: 10.1186/s40545-023-00592-7 (PMC10332012; doi:10.1186/s40545-023-00592-7)
Supplement: Supplementary file 1 — Additional file 1. Advanced Competencies for Hospital Pharmacists. [file 40545_2023_592_MOESM1_ESM.pdf]

Advanced Competencies for Hospital Pharmacists

Dear pharmacist,

You are invited to participate in a survey about advanced competencies and skills acquired upon graduation of your highest degree related to your current field of work.

This study conducted by a group of academic researchers aims to determine the domains that need strengthening for an optimal-performing public health system.

Your participation in this study is voluntary and anonymous, and the information gathered in this 20-minute questionnaire will be treated confidentially. By completing it, you are consenting to participate in this study.

We thank you in advance for your time,

The research team.

Informed consent

Please check all the boxes to proceed to the survey

☐ I have read and understood the above information

☐ I understand that my participation is voluntary

☐ I understand that my data will be kept confidential

☐ I agree to participate in this study

DEMOGRAPHICS

1. **Age:**
2. **Gender:** ☐M ☐F
3. **Level of education:**
   ☐BS Pharmacy ☐PharmD/DPharm ☐Masters ☐PhD ☐Other:
4. **Highest degree related to your main field of work:**
   ☐BS Pharmacy ☐PharmD/DPharm ☐Masters ☐PhD ☐Other:
5. **Year of graduation from school/faculty of pharmacy:**
6. **University you graduated from as a pharmacist:**
   ☐UL ☐USJ ☐BAU ☐LAU ☐LIU ☐Other, country:
7. **University you earned your highest degree from:**
   ☐UL ☐USJ ☐BAU ☐LAU ☐AUB ☐LIU ☐Other, country:
8. **Language of pharmacy education:**
   ☐French ☐English ☐Other:
9. **Work Location:**
   ☐Beirut ☐Mount Lebanon ☐North Lebanon ☐South Lebanon ☐Beqaa
   ☐Currently not working
10. **Number of working days per week:**
11. **Number of working hours per day:**
12. **Number of beds at the hospital (hospital/clinical pharmacists only):**
    ☐<50 ☐50-100 ☐101-300 ☐>300
13. **How long (in years) have you been practicing as a hospital pharmacist?**
14. **Do you have another field of work? (Please select all that apply)**
    ☐ I do not have another field of work
    ☐Academia (teaching)
    ☐Preceptor
    ☐Clinical pharmacy
    ☐Research
    ☐Other:

HOSPITAL PHARMACIST COMPETENCIES

QUESTION: How confident are you in applying the below hospital pharmacist competencies?	Very confident	Fairly confident	Neither/ I don't know	Slightly confident	Not confident at all
**0 Fundamental Skills**	**0.1 Regulations**				
**0.1.1** Apply pharmaceutical statutory regulations.					
**0.1.2** Follow hospital regulations pertaining to the operations of the hospital pharmacy.					
**0.1.3** Ensure prescriptions meet national legal requirements for medicines and medicinal products.					
**0.1.4** Ensure medicines are labelled appropriately and comply with legislation and national guidance.					
**0.1.5** Demonstrate knowledge of the employment laws.					
**0 Fundamental Skills**	**0.2 Drug Procurement & Management**				
**0.2.1** Select medications and develop a therapeutic formulary according the international and national guidelines.					
**0.2.2** Supervise procurement systems.					
**0.2.3** Ensure drug inventory management, including tracking of expiry dates, production lot numbers, and storage conditions.					
**0.2.4** Manage backorders and recalls.					
**0.2.5** Handle drug waste.					
**0.2.6** Handle drug shortages and suggest suitable alternatives of medicines and medical devices in a timely manner.					
**0.2.7** Acquire negotiation skills for drug procurement					
**0.2.8** Learn the complexity of the drug and medical supplies market					
**0.2.9** Consider cost-effectiveness when purchasing and dispensing stock and advising on prescribing choices.					
**0 Fundamental Skills**	**0.3 Optimize Processes Related to Organization, Medication Preparation, and Delivery**				
**0.3.1** Demonstrate organizational skills and develop standard operating procedures related to storage, safety, prescription, preparation, transcription, dispensing, administration, and monitoring steps.					
**0.3.2** Describe the appropriate roles of pharmacy staff and pharmacists in these processes.					
**0.3.3** Supervise pharmacy staff in their work in medication preparation and delivery.					

0 Fundamental Skills	0.4 Aseptic Techniques				
**0.4.1** Follow aseptic and sterilization techniques and describe processes and facilities needed to provide sterile compounded parenteral solutions, including the basic requirements of hospital accreditation standards.					
**0.4.2** Develop standards and maintain written procedures for sterile and aseptic production of medicines, and total parental nutrition.					
**0.4.3** Apply knowledge of hospital hygiene and infection prevention control (IPC).					
**0 Fundamental Skills**	**0.5 Pharmaceutical and Hospital Technology/Automation**				
**0.5.1** Outline the basic functionality of commonly used automated systems related to medication use (such as automated dispensing cabinets, computerized prescriber order entry systems, bar code, medication administration systems, programmable infusion devices, and robotics).					
**0.5.2** Understand their appropriate and safe use as well as unintended consequences.					
**0.5.3** Ensure the effective introduction of new technologies.					
**0 Fundamental Skills**	**0.6 Pharmaceutical/Medical Skills**				
**0.6.1** Implement the appropriate use of injectable medications, including intravenous, intrathecal, intraocular, intradermal, and other routes.					
**0.6.2** Formulate, reconstitute and compound medicines when needed.					
**0.6.3** Develop protocols for ensuring quality of prepared medicines.					
**0.6.4** Appraise the inter-relationships between formulation (including excipients), drug delivery and therapeutic product.					
**0.6.5** Integrate and interface the clinical and distributive functions, including the synergy that translates into safe and effective medication therapy.					
**0.6.6** Participate in designing and implementing pharmaceutical/therapeutic protocols and algorithms					
**0.6.7** Integrate pharmaceutical oncology, nutrition, and other fields when applicable.					
**0.6.8** Demonstrate knowledge of pharmaceutical radiotherapy: therapeutic and diagnostic applications (e.g., contrasts) when applicable.					
**0.6.9** Describe the use of medical devices, prostheses, and implants when applicable.					
**0 Fundamental Skills**	**0.7 Business Management Skills**				
**0.7.1** Perform accounting activities.					
**0.7.2** Perform financial management.					
**0.7.3** Set budgeting proposals					

**0.7.4** Manage the payment terms and schedules with the suppliers					
**1 Safe and Rational Use of Medicines**	**1.1 Patient Safety**				
**1.1.1** Understand patient safety culture that relates to medication use, pharmaceutical care, and pharmacist's role.					
**1.1 2** Reconcile effectively the medications of a patient transitioning from one care setting to another and make appropriate communications to involved pharmacy providers.					
**1.1.3** Manage the problems related to switch patient's medication to formulary medicines.					
**1.1.4** Employ performance improvement techniques used in health systems and describe how they are used to appropriate communications to pharmacy providers involved.					
**1.1.5** Describe the impact of pharmacist involvement on medication safety and quality using appropriate literature.					
**1.1.6** Implement and promote pharmacovigilance activities within the hospital					
**1.1.7** Report and document patient safety incidents.					
**1 Safe and Rational Use of Medicines**	**1.2 Quality Assurance**				
**1.2.1** Identify storage conditions and secure the medicines cold chain conditions.					
**1.2.2** Discuss with and advise healthcare providers on issues related to pharmaceutical products and quality standards.					
**1.2.3** Describe the quality standard of the accreditation bodies					
**1.2.4** Apply national standards, guidelines, best practices, and established principles and processes related to quality and safe medication use (e.g., storage of look-alike/sound-alike medications, high alert medications, storage of concentrated potassium in patient care areas, dangerous abbreviations, leading decimal points and trailing zeros, quality measures related to medications, etc.).					
**1 Safe and Rational Use of Medicines**	**1.3 Pharmacovigilance**				
**1.3.1** Plan and implement medicines management actions to minimize the risk related to these medicines.					
**1.3.2** Identify a potential adverse drug reaction					
**1.3.3** Consider that reporting an ADR is part of pharmacist duties					
**2 Patient-Centered Care**	**2.1 Literature Evaluation/Search/Trials**				
**2.1.1** Access appropriate drug information resources, including primary literature.					
**2.1.2** Read recently published studies critically					
**2.1.3** Provide accurate and evidence-based answers.					
**2.1.4** Develop and implement methods for clinical trials and observational studies.					
**2 Patient-Centered Care**	**2.2 Pharmacokinetic-Based Assessment**				

**2.2.1** List the medications that need pharmacokinetic evaluation.					
**2.2.2** Apply dosing principle.					
**2.2.3** Evaluate drug-response and monitor patients.					
**2.2.4** Evaluate medication-use patterns in a specified patient population.					
**2 Patient-Centered Care**	**2.3 Drug Use Optimization**				
**2.3.1** Demonstrate an appropriate level of clinical knowledge related to medications and therapeutics in making decisions or recommendations related to the clinical use of drugs when appropriate.					
**2.3.2** Contribute to the establishment of medication use policies, including anti-microbial stewardship, criteria, and maintenance of the formulary as a member of the Pharmacy and Therapeutics Committee.					
**2.3.3** Provide quality care through the best use of resources.					
**2.3.4** Draft and distribute information and recommendations.					
**2.3.5** Optimize use of drugs, including addition, deletion, dose adjustment, intravenous to oral route switch, renal dosing, dose reduction, etc.					
**3 Professional Skills**	**3.1 Written and Oral Communication**				
**3.1.1** Demonstrate effective verbal and written communications.					
**3.1.2** Communicate with pharmacy and healthcare team members.					
**3.1.3** Respond to questions with the appropriate level of detail necessary to ensure proper patient care and communication with other relevant parties.					
**3.1.4** Develop and maintain appropriate therapeutic recommendations related to medication therapy.					
**3 Professional skills**	**3.2. Professional advancement**				
**3.2.1** Engage in regular professional development activities					
**3.2.2** Engage in professional organization activities					
**3 Professional Skills**	**3.3 Behavior & Ethical**				
**3.3.1** Demonstrate professional behavior (attitude, dress, appearance, etc.) in practice settings.					
**3.3.2** Apply ethical principles.					
**3.3.3** Support staff in their professional and personal development.					
**3.3.4** Be able to carry out staff appraisals.					
**3 Professional Skills**	**3.4 Management**				
**3.4.1** Demonstrate project and team management skills.					
**3.4.2** Implement the medication management strategy or plan.					
**3.4.3** Plan and manage physical and financial resources.					
**3.4.4** Prioritize multiple patient care and triage in times of high activity and workload.					

**3.4.5** Demonstrate efficient problem-solving skills.					
**4 Pharmacist Emergency Preparedness and Response (EPR)**	**4.1 Emergency Preparedness and Response**				
**4.1.1** Check for volunteering opportunities					
**4.1.2** Check for training opportunities					
**4.1.3** Address medication shortage and mitigation plan					
**4.1.4** Balance stockpile and availability of drugs for existing/chronic conditions					
**4.1.5** Partner with local authorities					
**4.1.6** Check for FDA/EMA Emergency Use Authorizations (EUAs) and expedited review and approval of tests/drugs for treatment					
**4.1.7** Follow actions and recommendations of local authorities					
**4.1.8** Involve trainees and staff in emergency response					
**4 Pharmacist Preparedness and Response in Emergency Situations**	**4.2 Operations Management**				
**4.2.1** Procure essential medications and supplies					
**4.2.2** Ensure medication delivery/safe storage					
**4.2.3** Develop workplace training and safety protocols (e.g., social distancing)					
**4.2.4** Secure personal protective equipment or other needed materials					
**4.2.5** Monitor workers/assistants for symptoms					
**4.2.6** Adapt working hours to meet essential services during crises					
**4.2.7** Secure sanitizers and other medications when needed					
**4.2.8** Participate in interdisciplinary training to emergency preparedness and response teams					
**4 Pharmacist Preparedness and Response in Emergency Situations**	**4.3 Patient Care and Population Health Interventions**				
**4.3.1** Maintain patient confidentiality					
**4.3.2** Continue medication reviews, screening and/or testing/vaccination services safely					
**4.3.3** Identify at-risk populations					
**4.3.4** Educate peers about the ongoing crisis using evidence-based information and communications					
**4.3.5** Answer EPR-related calls					
**4 Pharmacist Preparedness and Response in Emergency Situations**	**4.4 Evaluation, Research, and Dissemination for Impact and Outcomes**				
**4.4.1** Participate in research and studies on EPR					
**4.4.2** Publish and/or disseminate findings					
**4.4.3** Combat misinformation by disseminating evidence-based information to patients and sharing it on social media					
**4.4.4** Develop training programs to peers and other healthcare workers					

1. **What percentage of these competencies did you acquire during your undergraduate studies?**
2. **What percentage of these competencies did you acquire during your postgraduate studies?**
3. **What percentage of these competencies did you acquire from continuing education sessions?**
   What percentage of these competencies did you acquire by experience?
