# Supplementary material for: Validation of the specialized competency framework for pharmacists in hospital settings (SCF–PHS): a cross-sectional study
Source: J Pharm Policy Pract. 2023 Jul 10;16:86. doi: 10.1186/s40545-023-00592-7 (PMC10332012; doi:10.1186/s40545-023-00592-7)
Supplement: Supplementary file 2 — Additional file 2. Advanced Competencies for Clinical Pharmacists. [file 40545_2023_592_MOESM2_ESM.pdf]

Advanced Competencies for Clinical Pharmacists

Dear pharmacist,

You are invited to participate in a survey about advanced competencies and skills acquired upon graduation of your highest degree related to your current field of work.

This study conducted by a group of academic researchers aims to determine the domains that need strengthening for an optimal-performing public health system.

Your participation in this study is voluntary and anonymous, and the information gathered in this 20-minute questionnaire will be treated confidentially. By completing it, you are consenting to participate in this study.

We thank you in advance for your time,

The research team.

Informed consent

Please check all the boxes to proceed to the survey

☐ I have read and understood the above information

☐ I understand that my participation is voluntary

☐ I understand that my data will be kept confidential

☐ I agree to participate in this study

• Corniche Al Nahr, Pierre Gemayel str, Pharmacists Funds bldg. • P.O. Box: 11-2807 Beirut - Lebanon •
• Phone: +961 1 611081 • Fax: +961 1 425247 • E-mail: opl@opl.org.lb •

DEMOGRAPHICS

1. **Age:**
2. **Gender:** ☐M ☐F
3. **Level of education:**
   ☐BS Pharmacy ☐PharmD/DPharm ☐Masters ☐PhD ☐Other:
4. **Highest degree related to your main field of work:**
   ☐BS Pharmacy ☐PharmD/DPharm ☐Masters ☐PhD ☐Other:
5. **Year of graduation from school/faculty of pharmacy:**
6. **University you graduated from as a pharmacist:**
   ☐UL ☐USJ ☐BAU ☐LAU ☐LIU ☐Other, country:
7. **University you earned your highest degree from:**
   ☐UL ☐USJ ☐BAU ☐LAU ☐AUB ☐LIU ☐Other, country:
8. **Language of pharmacy education:**
   ☐French ☐English ☐Other:
9. **Work Location:**
   ☐Beirut ☐Mount Lebanon ☐North Lebanon ☐South Lebanon ☐Beqaa
   ☐Currently not working
10. **Number of working days per week:**
11. **Number of working hours per day:**
12. **Number of beds at the hospital:**
    ☐<50 ☐50-100 ☐101-300 ☐>300
13. **How do you provide direct patient care activities?**
14. **How long (in years) have you been practicing as a clinical pharmacist?**
15. **Do you have another field of work: (Please select all that apply):**
    ☐I do not have another field of work
    ☐ Academia (teaching)
    ☐Clinical preceptor
    ☐Research
    ☐Other:

CLINICAL PHARMACIST COMPETENCIES

QUESTION: How confident are you in applying the below clinical pharmacist competencies?	Very confident	Fairly confident	Neither/ I don't know	Slightly confident	Not confident at all
**0 Quality Improvement**	**0.0 Medication Use Management**				
**0.0.1** Identify opportunities for improvement of the institution's medication-use system.					
**0.0.2** Understand the institution's medication-use system and its vulnerabilities to adverse drug events (ADEs).					
**0.0.3** Understand the impact of pharmacist involvement on medication safety and quality using appropriate literature.					
**0.0.4** Understand the structure and process of the medication-use system.					
**0.0.5** Participate in opportunities for improvement in the institution's medication-use system by comparing the medication-use system to relevant best practices.					
**0.0.6** Implement quality improvement changes to the institution's medication-use system.					
**0.0.7** Understand the process for developing, implementing, and maintaining a formulary system.					
**0.0.8** Document appropriate therapeutic recommendations related to medication therapy.					
**0.0.9** Make a medication-use policy recommendation based on a comparative review, e.g., ~~drug class review, drug monograph,~~ antimicrobial stewardship, etc.					
**0.0.10** Draft and distribute information and recommendations related to the clinical use of drugs when appropriate.					
**0.0.11** Participate in the identification of need for, development of, implementation of, and evaluation of an evidence-based treatment guideline/protocol related to individual and population-based patient care.					
**0.0.12** Participate in pilot interventions to change problematic or potentially problematic aspects of the medication-use system with the objective of improving quality.					
**0.0.13 Use clinical pharmacy metrics/indicators** to show the impact of clinical pharmacy services					
**0 Quality Improvement**	**0.1 Medication Dispensing**				
**0.1.1** Prepare and dispense medications following existing standards of practice and the institution's policies and procedures.					

**0.1.2** Identify the appropriateness (rational and safe use) of a medication order before preparing or permitting the distribution of the first dose.					
**0.1.3** Follow the institution's policies and procedures to maintain the accuracy of the patient's medication profile.					
**0.1.4** Prepare medication using appropriate techniques and following the institution's policies and procedures.					
**0.1.5** Dispense medication products following the institution's policies and procedures.					
**0 Quality Improvement**	**0.2 Workplace Management**				
**0.2.1** Understand the effect of accreditation, legal, regulatory, and safety requirements on practice.					
**0.2.2** Understand the principles of financial management of a pharmacy department.					
**0.2.3** Evaluate the workload, organize the workflow, and check the accuracy of the work of pharmacy staff or others.					
**0.2.4** Use knowledge of the principles of change management to achieve organizational, departmental, and/or team goals.					
**1 Clinical Knowledge and Skills**	**1.0 Analytical Skills**				
**1.0.1** Provide concise, applicable, comprehensive, and timely responses to requests for drug information from patients and healthcare providers.					
**1.0.2** Formulate a systematic, efficient, and thorough procedure for retrieving drug information.					
**1.0.3** Determine relevant information to evaluate from all retrieved biomedical literature.					
**1.0.4** Evaluate the usefulness of biomedical literature gathered.					
**1.0.5** Formulate responses to drug information requests based on analysis of the literature.					
**1.0.6** Provide appropriate responses to drug information questions that require pharmacists to draw upon their knowledge base.					
**1.0.7** Assess the effectiveness of drug information recommendations.					
**1 Clinical Knowledge and Skills**	**1.1 Fundamentals of Clinical Knowledge**				
**1.1.1** Show knowledge of basic physical health examinations and laboratory tests and show ability to interpret and respond adequately to such data.					
**1.1.2** Understand normal organ anatomy and function, the effects of disease states that affect medicines use.					
**1.1.3** Apply knowledge of pathophysiology to specific therapeutic areas and to particular patient groups.					
**1.1.4** Demonstrate an appropriate level of clinical knowledge related to medications and therapeutics in making decisions or recommendations.					

**1.1.5** Describe and discuss the pharmacology and pharmacotherapy of drugs in routine use.					
**1.1.6** Understand how administration, drug distribution, drug elimination influences medicines outcomes.					
**1.1.7** Understand the scientific basis of different dosage formulations, and adjuvant compounds, their design and influence on the clinical efficacy of medicines.					
**1.1.8** Know the advantages and risks of the new formulations.					
**1.1.9** Discuss the importance of emerging technologies in pharmacology and pharmacotherapy.					
**1.1.10** Support the management of acute toxicity and advice on appropriate antidotes.					
**1.1.11** Identify pharmacotherapy-induced resistance and anti-microbial stewardship.					
**1 Clinical Knowledge and Skills**	**1.2 Patient Data Collection, Assessment, and Therapeutic Planning Skills**				
**1.2.1** Collect and organize all patient-specific information needed by the pharmacist to prevent, detect, and resolve medication-related problems and make appropriate evidence-based, patient-centered medication therapy recommendations as part of the interdisciplinary team.					
**1.2.2** Determine the presence of any of the following medication therapy problems in a patient's current medication therapy:					
**1.2.2.1** Medication used with no medical indication.					
**1.2.2.2** Patient has medical conditions for which there is no medication prescribed.					
**1.2.2.3** Medication prescribed inappropriately for a particular medical condition.					
**1.2.2.4** Immunization regimen is incomplete.					
**1.2.2.5** Current medication therapy regimen contains something inappropriate (dose, dosage form, duration, schedule, route of administration, method of administration).					
**1.2.2.6** Therapeutic duplication.					
**1.2.2.7** Medication to which the patient is allergic has been prescribed.					
**1.2.2.8** Adverse drug or device-related events or potential for such events.					
**1.2.2.9** Clinically significant drug-drug, drug-disease, drug-nutrient, or drug-laboratory test interactions or potential for such interactions.					
**1.2.2.10** Medical therapy has been interfered with by social, recreational, non-prescription, or non-traditional drug use by the patient					
**1.2.2.11** Patient not receiving the full benefit of prescribed medication therapy.					

**1.2.2.12** Problems arising from the financial impact of medication therapy on the patient.					
**1.2.2.13** Patient lacks understanding of medication therapy.					
**1.2.2.14** Patient not adhering to medication regimen.					
**1.2.3** Assess options available for problem solving, considering possible outcomes of any therapeutic action.					
**1.2.4** Use benefit-risk assessments for evaluating alternative treatment strategies.					
**1.2.5** Optimize use of drugs including: addition, deletion, dose adjustment, IV to Po switch, renal dosing, dose reduction, etc.					
**1.2.6** Use an organized collection of patient-specific information, summarize patients' healthcare needs.					
**1.2.7** Evaluate medication-use patterns in a specified patient population ~~(geriatrics, pediatrics, etc.).~~					
**1.2.8** Make a referral to the appropriate healthcare provider based on the patient's acuity and the presenting problem, when presented with a patient with healthcare needs that cannot be met by the pharmacist.					
**1.2.9** Design evidence-based therapeutic regimen (for medicine ~~and medical devices~~).					
**1.2.9.1** Identify therapeutic goals and design a patient-centered regiment that meets the evidence-based therapeutic goals established for a patient.					
**1.2.9.2** Integrate patient-specific information, disease and drug information, ethical issues, and quality of life issues.					
**1.2.9.3** Consider pharmacoeconomic principles: identify cost effective medicines and medical devices using valid and relevant pharmacoeconomic data.					
**1 Clinical Knowledge and Skills**	**1.3 Monitoring & Follow-Up Skills**				
**1.3.1** Design a patient-centered, evidence-based monitoring plan for a therapeutic regimen that effectively evaluates the achievement of the patient-specific goals.					
**1.3.2** When appropriate, initiate the patient-centered, evidence-based therapeutic regimen and monitoring plan for a patient according to the institution's policies and procedures.					
**1.3.3** Accurately assess the patient's progress toward the therapeutic goal(s).					
**1.3.4** Redesign a patient-centered, evidence-based therapeutic plan as necessary based on evaluation of monitoring data and therapeutic outcomes.					
**1 Clinical Knowledge and Skills**	**1.4. Medication safety and surveillance**				
**1.4.1** Identify high-risk medicines and high-risk administration of medicines relevant to the healthcare setting.					
**1.4.2** Plan and implement medicines management actions to minimize the risk related to these medicines.					

**1.4.3** Identify a potential adverse drug reaction					
**1.4.4** Consider that reporting an ADR is part of pharmacist duties					
**1.4.5** Demonstrate knowledge on reporting an adverse drug reaction to relevant authorities					
**1 Clinical Knowledge and Skills**	**1.5 Transition of Care & Reconciliation Skills**				
**1.5.1** Communicate pertinent pharmacotherapeutic information to the receiving healthcare professionals, when a patient is transitioning from one healthcare setting to another.					
**1.5.2** Ensure that accurate and timely medication-specific information reconciliation procedure regarding a specific patient reaches those who need it at the appropriate time.					
**1.5.3** Document, reconcile and update patient medication history and received interventions.					
**2 Soft Skills**	**2.0 Communication Skills**				
**2.0.1** Use effective patient education techniques to provide counseling to patients and caregivers, including information on medication therapy, adverse effects, compliance, appropriate use, handling, and medication administration.					
**2.0.2** Appropriately select direct patient-care activities for documentation.					
**2.0.3** Use effective communication practices when documenting a direct patient-care activity.					
**2.0.4** Explain the characteristics of exemplary documentation systems that may be used in the organization's environment.					
**2.0.5** Provide clear and concise consultations to other health professional					
**2 Soft Skills**	**2.1 Interdisciplinary Approach**				
**2.1.1** Demonstrate cooperative, collaborative, and communicative working relationships with members of interdisciplinary healthcare teams as appropriate.					
**2.1.2** Prioritize and manage daily activities to deliver appropriate patient-centered care to each patient.					
**2.1.3** Demonstrate collaborative professional pharmacist-patient relationships as appropriate.					
**2.1.4** Recommend or communicate a patient-centered, evidence-based therapeutic regimen and corresponding monitoring plan to other members of the interdisciplinary team and patients systematically, logically, accurately, and timely and secure consensus from the team and patient.					
**2 Soft Skills**	**2.2 Professionalism, Ethics, and Patient Advocacy**				
**2.2.1** Demonstrate pride in and commitment to the profession through appearance, personal conduct, and association membership.					
**2.2.2** Act ethically in the conduct of all job-related activities.					

**2.2.3** Demonstrate ownership of and responsibility for the welfare of the patient by addressing pharmacy-related patient care problems.					
**2.2.4** Respect the rights of patients in therapeutic decisions and assist in providing information to facilitate their decision.					
**2.2.5** Respect and maintain the individual's right to confidentiality.					
**2.2.6** Engage in regular professional development activities					
**2.2.7** Engage in professional organization activities					
**2 Soft Skills**	**2.3 Leadership and Self-Management**				
**2.3.1** Practice self-managed continuing professional development to improve the quality of own performance through self-assessment and personal change.					
**2.3.2** Understand various leadership philosophies that effectively support direct patient care and pharmacy practice excellence.					
**2.3.3** Support staff training activities.					
**2.3.4** Engage in regular professional development activities					
**2.3.5** Engage in professional organization activities					
**3 Ability to Conduct Clinical Research**	**3.0 Research Project Management Skills**				
**3.0.1** Suggest a feasible design for a practice-related project.					
**3.0.2** Describe, explain and discuss commonly used research methodologies.					
**3.0.3** Participate in pharmacy practice research and clinical research.					
**3.0.4** Secure any necessary approvals, including IRB, for one's design of a practice-related project.					
**3.0.5** Implement a practice-related project as specified in its design (clinical trials and observational studies).					
**3.0.6** Effectively present the results of a practice-related project.					
**3.0.7** Employ accepted manuscript style to prepare a final report of a practice-related project.					
**4 Ability to Provide Effective Education**	**4.0 Educational Skills**				
**4.0.1** Use effective educational techniques in the design of all educational activities.					
**4.0.2** Use skill in case-based teaching.					
**4.0.3** Use public speaking skills to speak effectively in large and small group situations.					
**4.0.4** Use knowledge of audio-visual aids and handouts to enhance the effectiveness of communications.					
**5 Use Information Technology to Make Decisions and Reduce Errors**	**5.0 Informatics Skills**				
**5.0.1** Explain security and patient protections such as access control, data security, data encryption, privacy regulations, as					

well as ethical and legal issues related to the use of information technology in pharmacy practice.					
**5.0.2** Exercise skill in elementary use of databases and data analysis software.					
**5.0.3 Use healthcare delivery systems and health informatics to optimize the care of individual p**atients and patient populations					
**5 Pharmacist Emergency Preparedness and Response (EPR)**	**5.1 Emergency Preparedness and Response**				
**5.1.1** Check for volunteering opportunities					
**5.1.2** Check for training opportunities					
**5.1.3** Address medication shortage and mitigation plan					
**5.1.4** Balance stockpile and availability of drugs for existing/chronic conditions					
**5.1.5** Partner with local authorities					
**5.1.6** Check for FDA/EMA Emergency Use Authorizations (EUAs) and expedited review and approval of tests/drugs for treatment					
**5.1.7** Follow actions and recommendations of local authorities					
**5 Pharmacist Preparedness and Response in Emergency Situations**	**5.2 Operations Management**				
**5.2.1** Procure essential medications and supplies					
**5.2.2** Ensure medication delivery/safe storage					
**5.2.3** Develop workplace training and safety protocols (e.g., social distancing)					
**5.2.4** Secure PPEs or other needed materials					
**5.2.5** Monitor workers/assistants for symptoms					
**5.2.6** Adapt working hours to meet essential services during crises					
**5.2.7** Secure sanitizers and other medications when needed					
**5.2.8** Participate in interdisciplinary training to EPR teams					
**5 Pharmacist Preparedness and Response in Emergency Situations**	**5.3 Patient Care and Population Health Interventions**				
**5.3.1** Maintain patient confidentiality					
**5.3.2** Continue medication reviews, screening and/or testing/vaccination services safely					
**5.3.3** Identify at-risk populations					
**5.3.4** Educate patient about the ongoing crisis using evidence-based information and communications					
**5.3.5** Manage panic buying					
**5.3.6** Answer EPR-related calls					
**5 Pharmacist Preparedness and Response in Emergency Situations**	**5.4 Evaluation, Research, and Dissemination for Impact and Outcomes**				
**5.4.1** Participate in research and studies on EPR					
**5.4.2** Publish and/or disseminate findings					

**5.4.3** Combat misinformation by disseminating evidence-based information to patients and sharing it on social media					
**5.4.4** Develop training programs to peers and other healthcare workers					

1. **What percentage of these competencies did you acquire during your undergraduate studies?**
2. **What percentage of these competencies did you acquire during your postgraduate studies?**
3. **What percentage of these competencies did you acquire during from continuing education sessions?**
4. **What percentage of these competencies did you acquire by experience?**
